# Clinical and pathologic findings of splenic tumours in four horses: a case series

**DOI:** 10.1186/s13620-026-00353-3

**Published:** 2026-07-20

**Authors:** Claudia Gilders, Pamela Kelly, Vivienne Duggan

**Affiliations:** School of Veterinary Medicine, University College Dublin, Belfield, Dublin 4, Ireland

**Keywords:** Equine, Spleen, Tumour, Lymphoma, Melanoma

## Abstract

**Background:**

Splenic tumours are rare in horses, with limited published literature consisting primarily of individual case reports and one hepato-splenic lymphoma case series. This case series analyses the clinical presentation, diagnostic findings and clinical outcomes of four cases of splenic neoplasia that presented to a university referral hospital over a three year period (2022–2025).

**Case descriptions:**

Presented are two cases of primary splenic lymphoma, and two cases of metastatic melanoma. Three cases (75%) presented with chronic, non-specific clinical signs including weight loss, colic and ventral oedema. One case (25%) presented acutely due to hypovolaemia secondary to splenic rupture. In all cases, splenic masses were identified on transabdominal ultrasonographic examination and definitive diagnosis was confirmed by post mortem histopathological examination.

**Conclusion:**

Equine splenic neoplasia typically presents with chronic non-specific clinical signs, similar to human splenic mass presentation, although acute presentation may occur following splenic rupture. The most common primary splenic tumour identified was lymphoma while melanoma was the predominant metastatic disease. Transabdominal ultrasonography was reliable for identifying splenic masses, although histopathological examination remains essential for definitive diagnosis. Whilst rare, clinicians could consider splenic neoplasia as a differential diagnosis of horses presenting with chronic weight loss and non-specific abdominal signs. The extent of splenic neoplasia and visceral metastasis is often underappreciated on ante-mortem investigation.

## Introduction

The spleen is the second largest lymphoid organ in the body. It is multifunctional with immunological, foetal haematopoesis, red cell storage and clearance, and iron storage roles. The equine spleen can contract via sympathetic stimulation of α-smooth muscle receptors in the splenic capsule which can transiently increase the circulating red blood cells by up to 50%, increasing oxygen carrying capacity and aerobic function, facilitating maximal exercise [1]. While splenic masses are uncommon in horses, both neoplastic and non-neoplastic masses have been reported. Differentials for splenic masses in horses include abscess, granuloma, haematoma and neoplasia [2, 3, 3, 4].

Published literature on equine splenic neoplasia is mainly limited to case reports and one case series. Splenic tumours are rare and represent < 1% of all diagnosed tumours [5]. Neoplastic lesions that have been reported in the horse include haemangiosarcoma, lymphoma and metastatic melanoma [3, 6–8]. Secondary metastasis is more common than primary splenic neoplasia. Lymphoma is the most common metastatic neoplasia within the abdominal cavity, with splenic masses of either primary or secondary origin present in 4–37% of lymphoma cases [9, 10]. Metastatic melanoma is the most common extra-abdominal cause of secondary splenic involvement [11]. Melanoma accounts for 6–15% of cutaneous neoplasia in horses, with a higher predisposition in older grey horses [11]. Metastasis is reported in 14% of melanoma cases, of which 57% involve splenic metastasis [7]. However, as cutaneous masses are infrequently submitted for histopathological evaluation, the true prevalence may be higher than currently recorded. The predisposition in grey horses has a genetic basis, the STX17 gene both induces grey coat colouration and enhances melanocyte proliferation, thereby increasing the risk of malignant transformation [12].

Haemangiosarcoma is rare in horses; however, in a series of 35 cases, 43% demonstrated splenic involvement, highlighting the fact that equine haemangiosarcoma can involve the spleen [6].

In dogs splenic neoplasia is most commonly of primary origin, in stark contrast to horses, with haemangiosarcoma being the most frequently diagnosed. A ‘double two-thirds rule’ is commonly applied in canine splenic neoplasia, in which two-thirds of cases are malignant and two-thirds of those are haemangiosarcoma [13]. In humans, splenic metastasis is considered uncommon with a prevalence of just 0.6% at autopsy [14], with isolated splenic lesions seen in 5.2% of cancer patients [15]. Primary malignant splenic neoplasms include mostly angiosarcoma and lymphoma [15]. The most common primary tumour is lung, breast, colorectal cancer and cutaneous malignant melanoma, with cutaneous melanomas having the highest rate of splenic metastasis. Secondary splenic neoplasia most commonly takes the form of carcinomas. Splenic metastasis usually occurs in the context of multi-visceral involvement [14, 16].

This case series aims to describe the history, clinical presentation, clinicopathological findings, diagnostic imaging, gross and histopathologic results of 4 horses with splenic tumours that presented to the equine referral hospital over a 3 year period.

### Case 1

A 7-year-old thoroughbred gelding that was noted to be dull and depressed on the morning of presentation and had been administered flunixin meglumine (1.1 mg/kg IV). Improvement was short-lived therefore he was referred to the hospital. The horse had no history of trauma or medical issues. On presentation the horse was dull and tachycardic with a heart rate of 76 bpm and a tachypnoea of 44 breaths per minute. The horse was in good body condition with a score of 4/9 (Henneke [17]). The horse had pale, grey and tacky mucous membranes and a weak and thready pulse. During the examination the horse began to sweat, became distressed and was kicking at the abdomen.

On transabdominal ultrasonographic examination a marked amount of free peritoneal fluid was seen which was echogenic with a swirling, smoky appearance suggestive of blood. The stomach was normal sized and the small and large intestines appeared within normal limits with no thickening of the walls and good motility. The liver appeared to be within normal limits. The spleen was seen to have a large multi-lobulated mass associated with it, extending from the 7th intercostal space to the nephrosplenic space and extending caudo-ventrally beyond the costochondral junctions. The kidney was difficult to visualise with the mass obscuring the image.

On haematological and biochemical analysis the horse was mildly anaemic with packed cell volume (PCV) and total protein 26% (rr 29–44%) and 76 g/L (rr 57–79 g/L) respectively. Blood gas showed a metabolic acidaemia of 7.29 (rr 7.35–7.45) with marked hyperlactaemia of 6.2 mmol/L (rr < 2 mmol/L). The horse had a monocytosis of 1.07 × 10^9/L (rr 0.04–0.4 × 10^9/L) and a hyperfibrinogenaemia of 3.1 g/L (rr 0–2 g/L). An abdominocentesis yielded a haemorrhagic sample with a PCV of 19% (rr < 1%), lactate concentration of 17.5 mmol/L (rr < 2 mmol/l). Total protein and cell count were not available. Due to the clinical presentation and diagnostic imaging findings it was suspected that the horse was in hypovolaemic shock due to acute intra-abdominal haemorrhage. An intravenous catheter was placed and the horse was administered a bolus of crystalloid fluids of 10 ml/kg/hr and tranexamic acid (25 mg/kg) intravenously. The horse’s heart rate reduced to 60 beats per minute and PCV decreased to 19% over 12 h. Due to the poor prognosis of a likely neoplastic mass the owner elected for euthanasia.

**Fig. 1:**
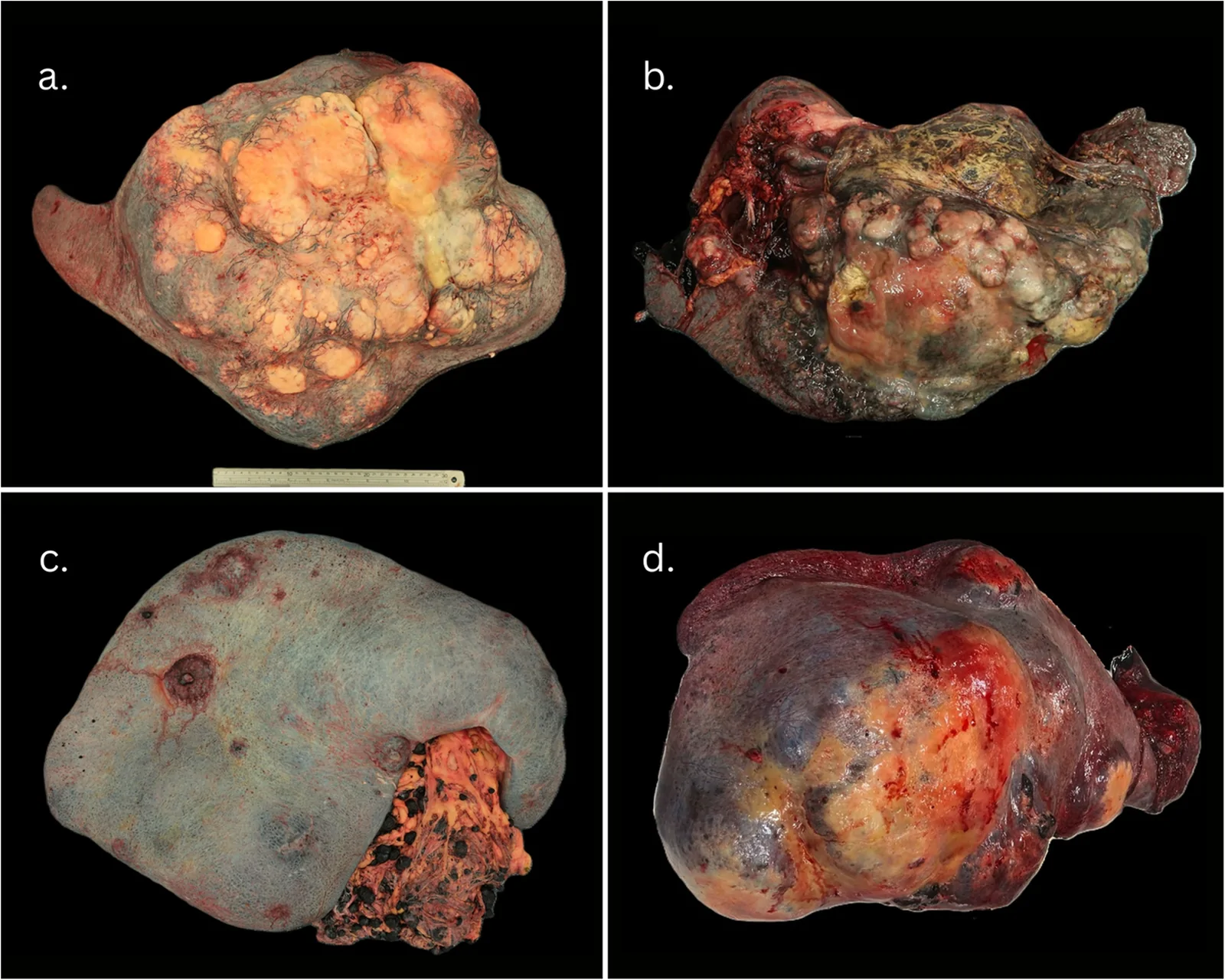
Gross post-mortem appearance of the spleen in cases 1(**a**), 2(**b**), 3(**c**) and 4(**d**)

A post mortem examination was performed which showed marked splenomegaly. The spleen weighed 19 kg (rr 2–4 kg). It had an irregular pale surface and a focal tear in the capsule which had resulted in the abdominal haemorrhage. There were multiple firm pale-yellow infiltrative lesions (Fig. 1a). On histopathology, the normal splenic architecture was effaced by an infiltrative mass of sheets of round cells that had large centrally placed round to ovoid nuclei featuring coarse chromatin stippling and minimal surrounding cytoplasm consistent with neoplastic lymphocytes. Mitotic index was 3–4 mitotic figures per high powered field (HPF or 0.237mm2). The cells were supported by a dense fibrous stroma that showed multiple areas of dystrophic mineralisation. There was no evidence of neoplasia in other organs including lymph nodes and bone marrow. Further immunohistochemical testing with markers for CD3, CD20 and CD79a showed that the neoplastic cells were diffusely and strongly positive for CD20 and CD79a indicating a B cell lymphoma. The horse was diagnosed with a primary splenic lymphoma with attendant splenomegaly, capsular rupture and haemoabdomen.

### Case 2

A 6-year-old sport horse gelding presented to the hospital for investigation of severe weight loss and inappetence over a 3 month period. The weight loss was initially mild but progressed rapidly in the 2 weeks prior to presentation. During this time the horse appeared to have a mild episode of colic which resolved with oral non-steroidal anti-inflammatories (NSAIDs). On presentation the horse was bright and alert but in a poor body condition, with a score of 3/9 (Henneke), weighing 542 kg with marked hind-limb and bilateral epaxial muscle atrophy. The horse had normal clinical parameters on clinical exam.

Haematologic and biochemical analysis was unremarkable apart from mild monocytosis (0.62 × 10^9/L) and hyper-fibrinogenaemia (3.08 g/L). An abdominal ultrasonographic examination was performed which showed splenomegaly; the spleen had a mixed echogenicity with nodular structures present throughout. There was a markedly irregular contour of the splenic margins. A marked amount of peritoneal fluid was also observed. The rest of the abdominal examination was within normal limits. Thoracic ultrasonography revealed mild bilateral pleural effusion. An abdominocentesis was performed and peritoneal fluid white blood cell count was within normal limits at 2.4 × 10^9/L (rr <5x10^9/L) but total protein concentration was elevated at 42 g/L (rr <20g/L). On abdominal palpation per rectum the spleen was displaced caudomedially. A large firm mass was palpable at the medial aspect of the spleen extending cranially. Gastroscopy performed under standing sedation revealed no abnormalities of the gastric mucosa. Due to the poor prognosis with a likely neoplastic process, the owner elected for euthanasia on welfare grounds.

**Fig. 2:**
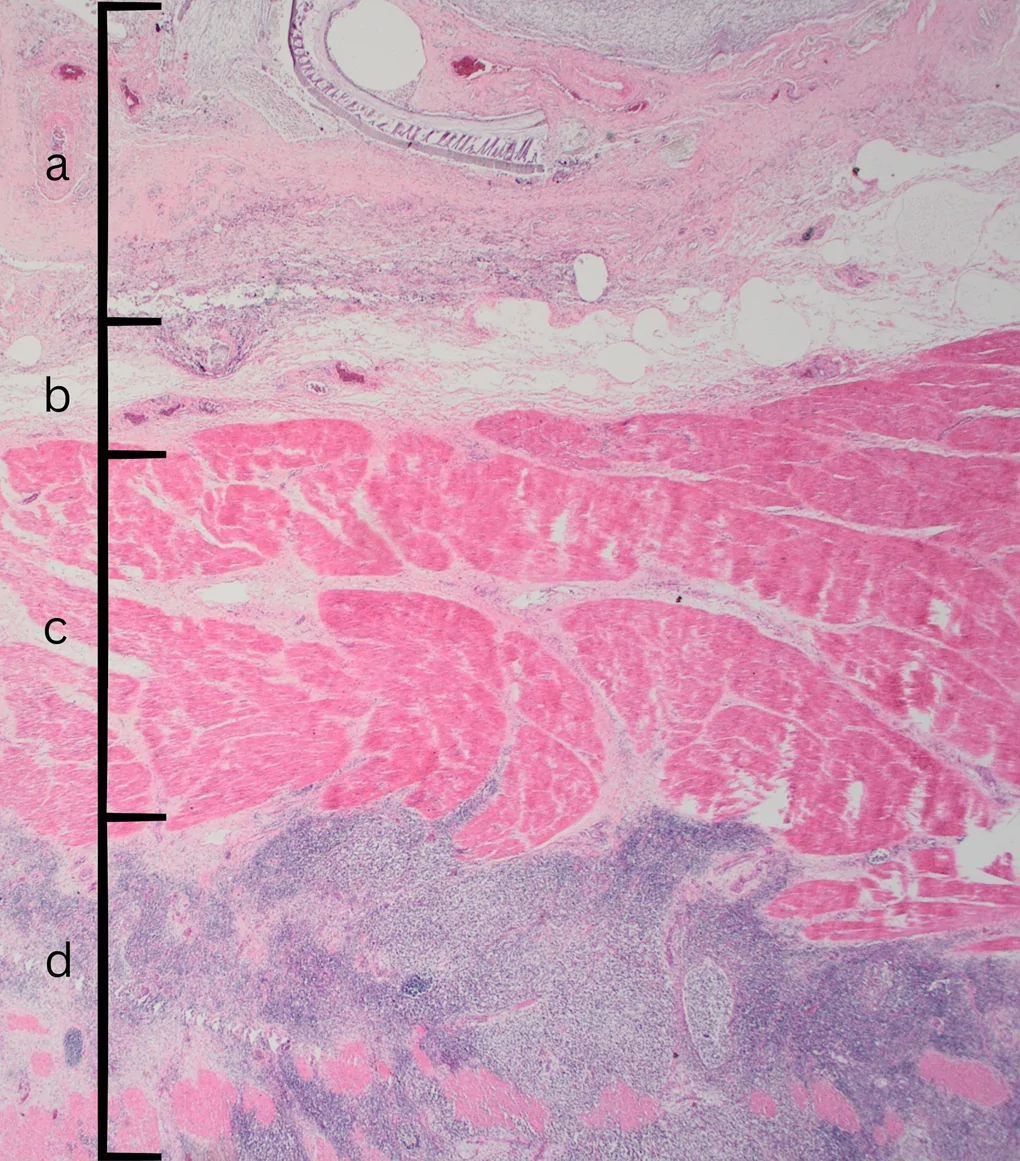
Microscopic image of stomach from case 2. The muscularis (**c**) and the serosa are infiltrated by sheets of neoplastic lymphocytes from the neoplastic mass (**d**). The mucosa (**a**) and submucosa (**b**) are autolytic but show no invasion of the neoplastic lymphocytes. Hematoxylin and Eosin

A post mortem examination showed marked splenomegaly; the spleen weighed 43.9 kg. Ninety percent of the splenic parenchyma was effaced with multifocal white nodules (Fig. 1b). Histologically the spleen was effaced by an infiltrative densely cellular, poorly demarcated, pseudo-encapsulated mass of round cells. These round cells were arranged in sheets and cords on a thin to moderately dense fibrovascular stoma. The cells had single round hyperchromic nuclei and showed moderate to high anisocytosis consistent with neoplastic lymphocytes. Miotic index was an average of 6 per HPF (0.237mm2). A large multi-nodular white mass similar to those found in the spleen was seen on the serosal side of the stomach at the level of the fundus, the liver showed multiple tan coloured foci and the iliac and ileocaecal lymph nodes were enlarged. Histologically the gastric mass (Fig. 2), and the changes seen in the liver and lymph nodes were due to infiltration of neoplastic lymphocytes consistent with that of the splenic mass. Given the gross size of the spleen, a diagnosis of primary splenic lymphoma with gastric, hepatic and lymph node metastasis was given. The neoplastic lymphocytes in the spleen were diffusely negative for CD3, CD20 and CD79a. However, throughout the mass were normal CD3 positive cells consistent with tumour infiltrating T cells which supports a diagnosis of T cell rich B cell lymphoma.

### Case 3

A grey 16-year-old Irish Sport Horse mare was referred to the hospital for an investigation of chronic colic and lameness. The horse had 3 episodes of mild colic over a period of three months; each episode resolved with medical treatment including NSAIDs and Buscopan Compositum administered by the referring veterinarian. The horse then developed a left forelimb lameness, which appeared to originate as a swelling at the medial elbow, the swelling then progressed down the limb and developed into a cellulitis with moderate swelling present from the elbow to the hoof. The horse had been treated with a course of TMPS (30 mg/kg PO BID) and phenylbutazone (2.2 mg/kg PO BID) to which there was minimal response; the swelling fluctuated but was deteriorating prior to presentation and the lameness was then evident at walk.

On arrival the horse was bright, alert and responsive, in good body condition, with a score of 4/9 (Henneke) and weighed 578 kg. Clinical parameters were within normal limits. The left forelimb had a larger focal swelling present at the medial aspect of the radius and moderate swelling distally. Serous discharge was present at the palmar aspect of the pastern proximal to the heel bulbs. There was mild heat associated with the limb swelling. The lameness was obvious at walk (grade 4/5 AAEP [18]).

Haematology and biochemistry showed a moderate increase in fibrinogen 2.6 g/L and an albumin: globulin reversal with hyperglobulinaemia 48.9 g/L (rr 24–48 g/L). There was a moderate leucocytosis from a neutrophilia 11.6 × 10^9/L (rr 2.2–5.8 × 10^9/L) and monocytosis 0.44 × 10^9/L. A mild increase was also seen in alkaline phosphatase 611 U/L(rr < 164U/L), glutamate dehydrogenase 33.3 U/L, (rr < 24U/L), aspartate aminotransferase 469 U/L, (rr < 416U/L) and creatine kinase 438 U/L, (rr < 91 U/L).

An abdominal ultrasonographic examination revealed multifocal circular masses throughout the spleen ranging in size from 3 cm-6 cm in diameter. Some masses appeared to have an anechoic centre. The spleen appeared to span the abdomen from the 4th rib to beyond the 18th rib suggestive of splenomegaly. No other masses or abnormalities could be identified. Abdominal palpation per rectum identified a large firm multi-lobulated mass to the left side of the caudal abdomen. The mass was not mobile and appeared to be adhered to the body wall close to the pelvic canal.

Due to the poor prognosis and likely neoplastic nature of the lesions the owner elected for euthanasia on welfare grounds. On post mortem examination multiple small nodular black pigmented masses were located in the pharynx, vulva, anus, ventral internal abdominal wall, diaphragm, lung, liver, retroperitoneal space and spleen. The spleen was enlarged, weighed 14 kg, had an irregular contour and contained multiple nodular masses (Fig. 1c). Histologically all masses were composed of packets and sheets of pigmented (melanin) cells that showed moderate to marked anisocytosis and anisokaryosis and there were large areas of necrosis. Mitotic index was an average of 3 per HPF (0.237mm2), however, the count varied considerably by lesion. The tumours within the perianal/perivulval skin had a low mitotic count (0–1 in 10 HPF), whereas the metastases showed progressively higher activity: the diaphragm/abdominal wall nodules 0–3 in 10 HPF, the splenic mass up to 3 per single HPF, the liver up to 3 in a single HPF, and the pulmonary, large intra-abdominal/intrapelvic and splenic masses the highest at 8 in 10 HPF.

**Fig. 3:**
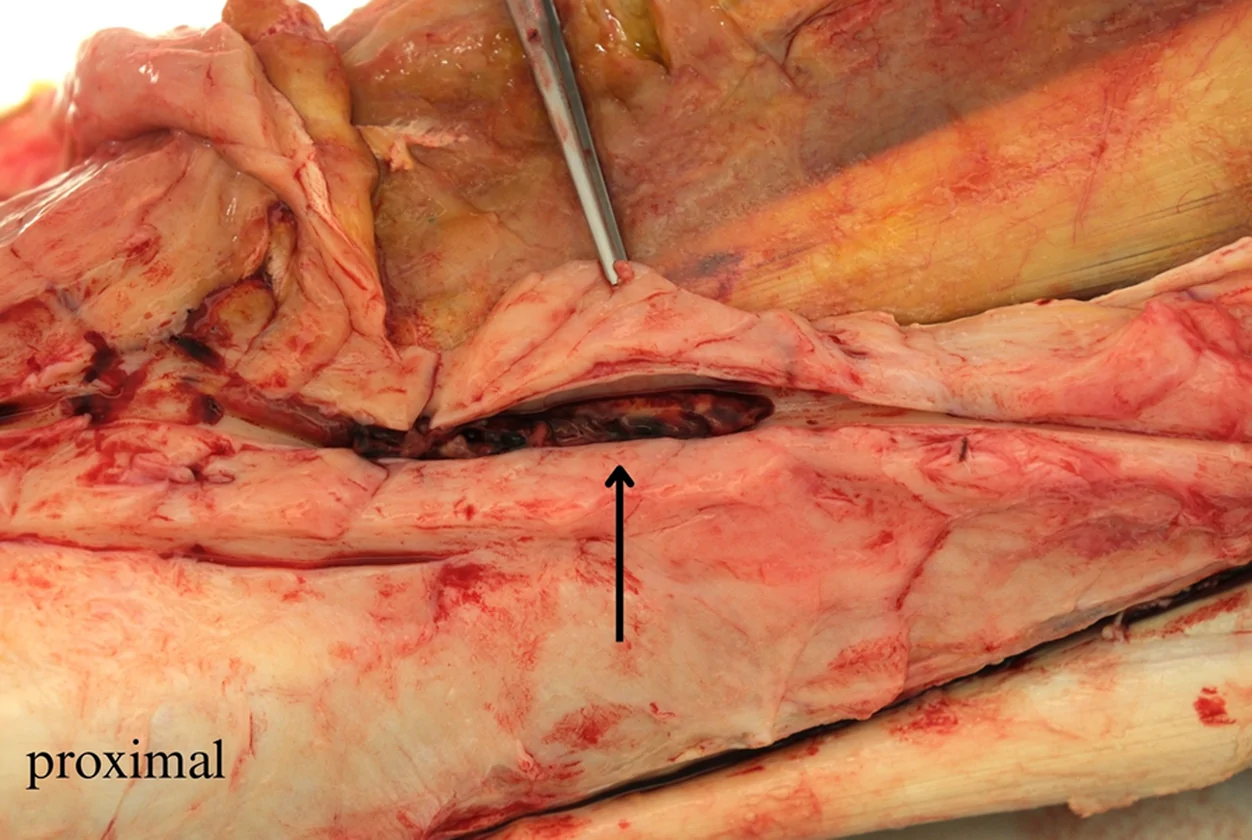
Thrombi present in the metacarpal vessel in case 3 highlighted by arrow

Gross examination of the left forelimb showed a severe, necrotizing cellulitis that was associated with thrombi within the medial and lateral metacarpal vessels (Fig. 3), ulceration and exudation of the coronary band and haemorrhage and necrosis of the hoof laminae consistent with acute laminitis. A swab of the thrombi was submitted for culture, but no growth occurred after 36 h. Histological examination of the musculature, vessels occluded by thrombi and hoof laminae confirmed an inflammatory process and showed no evidence of melanoma metastasis.

The horse was diagnosed with metastatic melanoma. The primary tumour in this case was thought to be the perianal/perivulval melanomas as the cells in these lesions appeared uniform and showed low mitotic activity. Whereas the cells in the other masses appeared more atypical with higher mitotic activity. Interestingly in this case, the melanoma found in the pharyngeal region; presumably within a lymph node, was monomorphic and had low mitotic activity similar to the perianal and peri-vulval skin tumours.

### Case 4

A 24-year-old grey Connemara gelding was referred to the hospital. It had been previously seen on three occasions. The initial visit was 11 years prior; it presented at that time for a mass under the 16th rib space on the right hand side of the abdomen. This was suspected to be a melanoma due to its ultrasonographic appearance; this mass did not change in size or appearance over subsequent visits. The horse re-presented for a second time 4 months prior to the final presentation for chronic sheath swelling and paraphimosis. On presentation, he had diffuse ventral oedema extending from the sheath cranial in the ventral abdomen. He was mildly tachycardic with a heart rate of 48 bpm; otherwise clinical parameters were within normal limits. Multiple small nodular masses were located in the parotid gland, tail base, sheath regions and displacing the anus. The mass under the16^th^ rib remained unchanged. All masses were suspected to be melanoma due to their appearance.

The paraphimosis was reduced with a ‘probang’ and cold hose therapy. An abdominal ultrasonographic examination showed multiple hyperechoic lobulated masses on the left side of the abdomen extending from the 5th intercostal space to the flank. The spleen had a heterogenous appearance with multiple circular lesions seen within. The liver had diffuse hyperechoic small circular lesions throughout. No free fluid was seen in the abdomen; no other abnormalities were noted in the abdomen. On abdominal palpation per rectum a grapefruit-sized mass was located at the anal sphincter which was firm and nodular. There was an additional multi-lobulated firm mass which extended dorsally in the position of the abdominal aorta as far cranially as could be palpated. The masses obstructed palpation of the spleen and left kidney. It was suspected due to their number and size that the masses could be obstructing lymphatic drainage and causing the sheath swelling. Haematologic and biochemical analysis revealed a mild anaemia with a packed cell volume of 29% and a hyper-fibrinogenaemia of 3.12 g/L. Although both albumin and globulin concentrations were within normal limits 32 g/L (rr 29–37 g/L) and 44 g/L (rr 24–48 g/L) respectively, there was a reversal of the normal ratio.

The owner was given a poor long term prognosis due to suspected metastatic melanoma. The owner was not ready to elect for euthanasia at this time so the horse was retired from all ridden work and continued with field turn out with strict monitoring for signs of discomfort, inappetence and weight loss. On final presentation, four months later, the horse had acutely decompensated. Body condition score had reduced from 5/9 to 3/9 with a weight loss of 68 kg. Urinary incontinence and non-weight bearing lameness (5/5 AAEP [18]) of the left hind limb had developed. No further work up was initiated, and the horse was euthanized on welfare grounds.

**Fig. 4:**
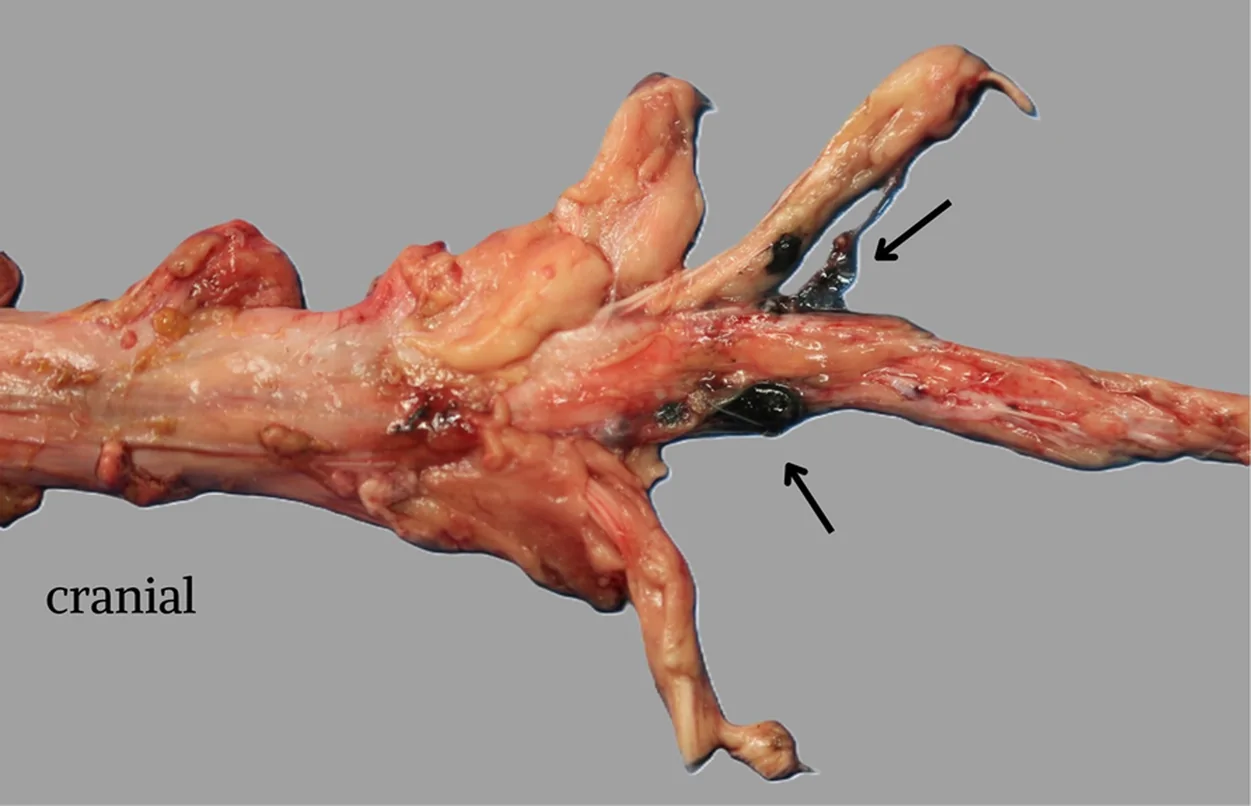
Melanoma present within the cauda equina in case 4 highlighted by arrows

Post mortem examination found multiple small (0.1 cm – 2 cm in diameter), nodular, black, cutaneous masses present around the perineum and anus which exuded black pigmented material when cut (melanin). Similar masses of variable size (0.1 cm – 25 cm in diameter) infiltrated and expanded the spleen (Fig. 1d), liver, mediastinal lymph nodes, epicardium, endocardium, coronary adipose tissue, mesentery, pleura, diaphragm, and extended from the caudal abdominal cavity through the pelvic canal into the perirectal and perineal regions. The lesion occupying the caudal abdomen incorporated the renal capsule (approximately 50 cm x 20 cm x 20 cm). Two small black nodular foci (approximately 5–10 mm in diameter) were also present within the cauda equina (Fig. 4). The abdomen also contained copious (approximately 10-20L) serosanguinous fluid.

Histopathological examination of the spleen showed a densely cellular, unencapsulated, infiltrative neoplasm composed of sheets and packets of polygonal to rounded to spindle-shaped cells supported by pre-existing fibrovascular stroma. Individual cells contained moderate to abundant eosinophilic cytoplasm, filled with variable, often abundant, intracytoplasmic brown to black granules (melanin), and had a round to irregular nucleus with finely stippled to coarsely clumped chromatin and 1–3 inconspicuous nucleoli. Anisocytosis and anisokaryosis were moderate. Miotic index was an average of 3–4 per HPF (0.237mm2). All other masses showed similar changes. In the cauda equina, within the perineural adipose tissue, were dense aggregates of neoplastic cells similar to those described in the spleen. The horse was diagnosed with metastatic melanoma. Given that the neoplastic cells within the cauda equina were present only in the perineural adipose tissue the neurological deficits were presumed to be from compression due to the highly expansile nature of the neoplasm.

## Discussion

The signalment, clinicopathological and investigatory findings are summarised in Table 1. The cases presented show a wide age range of 6- 24 years, with a mean age of 13 years and 3 months. Lymphoma cases occurred at a notably younger mean age of 7.5 years (range 7–8 years) compared to metastatic melanoma cases, which had a mean age of 20 years (range 16–24). This is consistent with the literature, as lymphoma has been reported across all ages with a median age of 5 years in one study [19]. Conversely, melanoma is more common in older horses, with a reported occurrence of up to 80% of older grey horses [20]; both metastatic melanoma cases in this series were older grey horses.

**Table 1:** Summary of clinical exam, clinical pathology and histopathology findings between cases 1–4

	Case 1	Case 2	Case 3	Case 4
Age	7	8	16	24
Breed	Thoroughbred	Sports Horse	Sports Horse	Connemara
Sex	Gelding	Gelding	Mare	Gelding
Colour	Bay	Bay	Grey	Grey
Use	Pleasure horse. Ex-Racehorse	Eventer	Pleasure horse	Pleasure horse
Reason for presentation	Dull and depressed, colic	Weight Loss	Chronic Colic and Cellulitis	Sheath Swelling and Paraphimosis
Vital signs	Tachycardia (76 bpm), tachypnoea, tacky grey mucous membranes	Within normal limits	Within normal limits	Tachycardia (48 bpm)
Haematology	Anaemia, acidaemia, monocytosis,	Monocytosis	Monocytosis, neutrophilia	Anaemia
Biochemistry	Hyperfibrinogenaemia	Hyperfibrinogenaemia	Hyperfibrinogenaemia, hyperglobulinaemia, A:G reversal, increased ALP, GLDH, AST and CK	Hyperfibrinogenaemia A:G reversal
Palpation per rectum abnormalities	Not performed	Y	Y	Y
Ultrasonography abnormalities	Y	Y	Y	Y
Outcome	Euthanised	Euthanised	Euthanised	Euthanised
Splenomegaly	Y	Y	Y	Y
Splenic weight	19 kg	43.9 kg	14 kg	Not performed
Tumour type	Lymphoma	Lymphoma	Melanoma	Melanoma
Mitotic figures	3–4	6	3	3–4
Primary/Secondary	Primary	Primary	Secondary	Secondary

Table Key: *Y* Yes, *A:G* Albumin:globulin, *ALP* Alkaline phosphatase, *GLDH* Glutamate dehydrogenase, *AST* Aspartate aminotransferase, *CK* Creatine kinase

The breed and sex distribution were representative of the hospital caseload. Presenting signs were mostly chronic in nature; however, one horse presented with acute decompensation of a chronic condition and another presented in acute circulatory crisis secondary to splenic rupture. Only the horse with the acute presentation due to haemorrhage had significantly abnormal clinical parameters, accompanied by severe haematological abnormalities. The remaining horses’ haematology and biochemistry profiles were consistent with nonspecific chronic inflammation. All 4 cases had hyperfibrinogenaemia (mean 2.9 g/L range 2.6–3.2 g/L) suggesting chronicity; two horses had an albumin to globulin (A:G) reversal, though only one was hyperglobulinaemic. Two horses were anaemic and 3 horses had monocytosis suggestive of chronic inflammation. One horse had neutrophilia, most likely attributable to the necrotic inflammatory lesion in the forelimb. Calcium concentrations were within normal limits in all cases, and no significant differences in haematologic findings were identified between melanoma and lymphoma cases.

Hyperfibrinogenaemia is the most commonly reported clinical chemistry abnormality in equines in lymphoma, occurring in 70% of cases [19] and was present in both lymphoma cases in this series with values exceeding 3 g/L. Hypoalbuminemia and hyperglobulinemia, also commonly seen with lymphoma were absent in both cases, possibly reflecting differences in organs, tissues or cells affected. This is in contrast to another study in which 2 of 4 (50%) hepatosplenic lymphoma cases were hyperglobulinaemic [10]. Hyperfibrinogenaemia, hyperglobulinaemia and an A:G reversal was noted in both cases of metastatic melanoma likely reflecting the advanced and chronic nature of disease at presentation. This pattern was not observed in a study of metastatic melanoma, in which elevations in liver enzyme activity were more prominent, likely due to organ involvement [7]. Only one melanoma case in this case series had elevated liver enzyme activity, but both cases had hepatic metastasis. Anaemia was noted in 2 cases, which except for the case of acute haemorrhage could be attributed to anaemia of chronic disease, increased erythrocyte destruction secondary to splenic congestion or immune-mediated disease arising from altered surface antigen exposure [11].

The 2 cases of metastatic melanoma presented for clinical signs were of unknown relation to underlying neoplastic masses. The relationship between the forelimb lesions and the disseminated melanoma in case 3 is uncertain. The extensive myonecrosis, fibrosis and necrotising cellulitis, together with thrombosis of the metacarpal vessels, are most consistent with a chronic, active inflammatory process, possibly of infectious (e.g. bacterial) aetiology with secondary vascular thrombosis. The thrombi likely compromised distal perfusion, producing the ischaemic laminar haemorrhage, necrosis and hoof separation. Although melanoma can metastasise to skeletal muscle, no neoplastic melanocytes were identified despite extensive dissection and sampling. A small, undetected metastasis cannot be excluded, but on the available evidence the limb lesions appear to represent a separate, possibly infectious, process rather than a direct manifestation of the melanoma. Therefore the initial reason for the lameness presentation was likely to be unrelated to the neoplasia identified. The chronic colic in comparison was suspected to be due to the splenomegaly.

In contrast to this in case 4, it was suspected that the 50 × 20x20cm mass extending from the caudal abdomen and through the pelvic canal could contribute to the sheath oedema due to lymphatic obstruction. The cause of the lameness was also unknown but suspected to be associated with this same mass obstructing on pelvic nerves or from the metastasis associated with the cauda equina. Marked splenomegaly was a consistent finding in all 4 cases; splenic weight recorded in three cases and ranged from 14–43.9 kg. Normal splenic weight, as a percentage of body weight, differs by breed, ranging from 0.19% in draft breeds to 0.3% in racing breeds [21]. In this series, splenic mass comprised 2.4–8% of body weight confirming severe splenomegaly in all cases.

Ultrasonographic examination has been previously described as a useful aid in diagnosing splenic masses of both non-neoplastic [4] and neoplastic origin [3]. In all four cases, abdominal ultrasonography revealed abnormal splenic architecture, with splenomegaly and splenic masses identified in all. Splenic masses are easily identifiable on ultrasonography due to the close approximation of the spleen to the body wall and its homogenous appearance [22]. Previously reported ultrasonographic findings in 3 cases of splenic neoplasia described a variety of appearances from heterogenous masses to generalised splenomegaly [3] consistent with the findings in this case series. However, ultrasonography alone cannot establish the aetiology of splenic masses [4].

Abdominal palpation was carried out in three cases, all of which resulted in detectable abnormalities. Gastroscopy was unable to identify the mass in the stomach in case 2 as it was located within the muscularis and serosa and did not extend to or disrupt the submucosa or mucosa, highlighting the limitations of some diagnostic imaging modalities. Although the marked splenomegaly in this case (43.9 kg) brought the spleen into contact with the stomach, the intramural (muscularis) location of the metastases, alongside abdominal lymph node involvement, favours lymphatic and/or haematogenous spread over direct transcoelomic seeding. Definitive diagnosis requires biopsy, performed either laparoscopically or percutaneously. In this case series no owners elected to proceed with antemortem diagnosis due to poor prognosis at presentation All ante-mortem investigations failed to identify the full extent of the neoplastic masses and metastasis found on post-mortem suggesting severity could be underestimated.

The splenic neoplasias identified consisted of lymphoma and metastatic melanoma. Both cases of lymphoma were primary splenic and suspected to be of B cell origin. The most common subtype of equine lymphoma is T-cell rich large B-cell lymphoma representing 47–55% of lymphoma cases [10, 23]. One lymphoma case had metastasised to the stomach, while the other had showed no signs of metastasis. Both melanoma cases were metastatic, with presumed cutaneous primary; however, given the extensive dissemination in both cases definitive identification of the primary site was not possible. Splenectomy has been reported with a successful outcome for a solitary splenic neoplasm in a mule [8]. In this case series, with the exception of one case of lymphoma which was confined to the spleen, the presence or extent of metastasis precluded this from being a possible treatment option.

Lymphoma is the most common haematopoietic tumour in horses representing 0.2–5% of all equine tumours [11]. It is most commonly categorised into four anatomical forms; multicentric, alimentary, mediastinal and cutaneous [9]. One previously reported case of splenic T cell-rich B cell lymphoma in a mule also presented with a haemoabdomen with similar histopathological findings; however, in that case the haemoabdomen was controlled conservatively and a splenectomy was performed [8]; an option that was not financially feasible for the owner in the present case. Another report described splenic lymphoma leading to the paraneoplastic syndrome of pseudo-hyper-parathyroidism and associated clinical signs of PU/PD [24]. Electrolyte concentrations were within normal limits in both lymphoma cases in this series.

For equine lymphoma, the WHO classification stratifies cases by mitotic index as follows: indolent = 0–1 mitoses, low grade = 2–5, mid-grade = 6–10, and high grade = > 10 [23]. Therefore the 2 lymphoma cases would be classified as low and mid-grade respectively. However, it is recognised that the primary tumour sites often have a lower mitotic index than metastatic lesions [25] therefore this could just reflect the primary nature of these tumours rather than a truly indolent biological behaviour. The acute nature in which some horses present with a haemoabdomen with splenic lymphoma is consistent with the clinical presentation commonly seen with splenic haemangiosarcoma in dogs, in which malignant causes account for 60–87% of haemoabdomen cases arising from splenic masses [13, 26]. Splenic rupture has also been reported, but less commonly, in humans; splenomegaly is reported in 92% of splenic angiosarcomas with 13% resulting in splenic rupture and haemoperitoneum, [27] rupture has also been reported in cases of splenic lymphoma [28].

Of the four recognised categories of equine melanoma, three carry malignant potential; dermal melanoma, dermal melanomatosis and anaplastic malignant melanoma. Based on the extent of metastasis and the clinical signs, both cases in this report would be classified as stage 5 equine malignant melanoma [12]. In both cases, a primary cutaneous melanoma is presumed to have undergone metastatic progression. Studies have found that disease duration correlates with increases in melanoma size and number but it is unclear if this is always direct metastasis from a singular site. Horses reportedly progress by 1 grade every 3 years [12]. Tumour size however corresponds poorly with histological classification, and even small masses should be considered as possible sites of metastasis [29].

Due to the high likelihood of metastasis with melanomas it is currently recommended to excise melanomas as early as possible [12]. The original presentation of case 4 was before the shift against ‘benign neglect’ had started to be widely recommended. A mass of that size and location would have presented a significant surgical challenge compared to cutaneous melanomas and wide margins were likely not possible. However with the advancement of chemotherapeutics and tyrosine kinase vaccinations this case would likely be approached differently now with the aim to at least reduce tumour size. It is also important to improve owner education to remove smaller new masses, as when the horse presented 11 years later there were melanomas visible in multiple sites which had passed the point of surgical excision. On the second presentation due to poor long term prognosis and extent of neoplasia, treatment was not performed and euthanasia was recommended.

There are two proposed theories to explain why the spleen is not a more common site of metastasis. The first is anatomical: the constant flow of blood and splenic contractions impede tumour cell adhesion, the acute angle of the splenic artery prevents larger tumour cell emboli from entering and the spleen has limited afferent lymphatics limiting lymphatic metastasis. The second theory proposes that cancer cells which reach the spleen are exposed to pro-apoptotic signals similar to those encountered by senescent erythrocytes and consequently fail to establish as metastases [16, 30].

## Conclusion

To conclude, splenic neoplasia in horses is rare. In this case series it represented approximately 0.006% of the cases seen by the internal medicine service over the three year period. Affected horses typically present with a chronic non-specific clinical profile, similar to that observed in humans with splenic masses, though acute presentation secondary to splenic rupture can occur. Splenic masses are easily identifiable with abdominal ultrasonography and are often detectable by abdominal palpation per rectum. In this series the most common primary splenic tumour was B-cell lymphoma while the most common secondary tumour was metastatic melanoma. Lymphoma cases occurred in younger horses, whereas metastatic melanoma cases were confined to older grey horses. The prognosis for splenic neoplasia is poor, and the true extent of metastatic disease is often underappreciated prior to post-mortem examination meaning splenectomy is a viable option only in rare cases of a solitary, non-metastatic splenic mass.

## Data Availability

No datasets were generated or analysed during the current study.
